# Trends in Underrepresented Race and Ethnicity Among Internal Medicine-Pediatrics Residents and Related Specialties: 2005-2020

**DOI:** 10.7759/cureus.78239

**Published:** 2025-01-30

**Authors:** Nicole Oakman, Kim Le, Joan Reisch, Jennifer B Walsh

**Affiliations:** 1 Internal Medicine-Pediatrics, University of Texas (UT) Southwestern Medical Center, Dallas, USA; 2 Internal Medicine, University of Texas (UT) Southwestern Medical Center, Dallas, USA; 3 Internal Medicine, Icahn School of Medicine at Mount Sinai, New York, USA; 4 Peter O’Donnell Jr. School of Public Health, University of Texas (UT) Southwestern Medical Center, Dallas, USA

**Keywords:** diversity and inclusion, family medicine residency, graduate medical education (gme), internal medicine & pediatrics, internal medicine residency, medicine-pediatrics, med-peds, med-peds residency, pediatric residency, underrepresented racial/ethnic minorities in medicine

## Abstract

Background

Increased diversity of race and ethnicity in internal medicine-pediatrics (med-peds) and related specialties may improve health disparities in the United States (US). Family medicine (FM), internal medicine (IM), and pediatric residencies have demonstrated variable and inconsistent trends in the representation of underrepresented minorities in medicine (URiM), but information about URiM med-peds trainees is limited. The primary aim was to compare trends in URiM representation from 2005 to 2020 among US med-peds residents to FM, IM, and pediatrics. The secondary aim was to compare these trends to the US population.

Methods

Self-reported race and ethnicity data was obtained from annual graduate medical education census reports and the US Census Bureau. A cross-sectional analysis of African American/Black (AA), American Indian/Alaskan Native, Native Hawaiian/Pacific Islander, and Hispanic representation among the US population and med-peds, FM, IM, and pediatric residents was performed. Trends in proportions of URiM representation among residents and the US population were evaluated using the Cochran-Armitage test.

Results

There was a significant positive trend in the proportion of total URiM residents in med-peds (11.0% (n = 156) to 15.1% (n = 226), p = 0.01), FM (16.5% (n = 1,550) to 18.7% (n = 2,565), p = 0.04), IM (12.3% (n = 2,688) to 15.5% (n = 4,455), p < 0.001), and pediatrics (14.9% (n = 1,187) to 18.4% (n = 1,670), p < 0.001). Total URiM med-peds representation remained below other specialties throughout the study. Med-peds residencies demonstrated a significant positive trend in Hispanic representation (3.3% (n = 47) to 7.7% (n = 116), p < 0.001) and a significant negative trend in AA representation (6.9% (n = 98) to 7.1% (n = 107), p = 0.01). Total URiM representation remained below the US population for all specialties throughout the study.

Conclusion

The total proportion of URiM med-peds, FM, IM, and pediatric residents improved. For med-peds, this improvement was largely due to increased Hispanic resident representation. However, URiM med-peds resident representation is consistently below related specialties. URiM resident representation in med-peds and related specialties poorly reflects the diversity of the US population and should be urgently addressed.

## Introduction

Despite efforts to improve healthcare accessibility and delivery, racial and ethnic minority populations continue to experience significant health disparities across the age spectrum from pediatric to adult care [1-3]. Creating a diverse physician workforce representative of the United States (US) population may help reduce health inequality by increasing access to care and improving patient-physician relationships [4]. Non-White physicians disproportionately provide care to underserved populations [5]. Patient-physician racial concordance is associated with better patient communication and improved health outcomes, such as decreased mortality for Black infants [4,6-9]. Patient-physician racial concordance is also associated with lower emergency department utilization and hospitalization rates for Hispanic adults, as well as decreased healthcare expenditure for both Black and Hispanic adults [10]. While individuals identifying as African American/Black (AA), American Indian/Alaskan Native (AI/AN), Native Hawaiian/Pacific Islander (NH/PI), and Hispanic comprised more than 30% of the US population in 2018, the Association of American Medical Colleges (AAMC) reported these groups represented <15% of active physicians and are underrepresented minorities in medicine (URiM) [11]. In recent years, the Accreditation Council for Graduate Medical Education (ACGME) and the AAMC have called for additional efforts to increase racial and ethnic diversity in residency programs [11,12].

Internal medicine-pediatrics (med-peds) residents complete a four-year training program equally divided between internal medicine and pediatrics and are eligible for board certification in both specialties. They are uniquely positioned to address health disparities impacting all ages and to impact health outcomes for young adults transitioning from pediatric to adult care, such as patients with sickle cell disease, who experience increased mortality during this transition [13]. A significant number of med-peds graduates pursue general practice, with approximately 33% entering ambulatory primary care, 21% pursuing hospital medicine, and up to 21% practicing in rural or underserved areas [14,15]. Medical students applying to med-peds residency often consider applying to specialties that provide comparable clinical training and career opportunities, including family medicine (FM), internal medicine (IM), and pediatrics [16,17]. Thus, comparing trends in URiM resident representation across med-peds and these overlapping specialties may be useful.

Prior research suggests variable improvement in racial and ethnic diversity within FM, IM, and pediatrics [18-20]. There have been substantial gains in racial and ethnic diversity within FM, but AA and Hispanic residents continue to be underrepresented compared to the US population [18,21]. Liao et al. noted a minimal but statistically significant increase in URiM IM resident representation between 2010 and 2018 [20]. Montez et al. found no significant upward trend in URiM representation among pediatric residents from 2007 to 2019 [19]. Research about trends in racial and ethnic diversity among med-peds residents is limited. In addition, each of these studies utilized different statistical methods and date ranges, which impedes the ability to compare trends in URiM representation between specialties.

The primary aim of this study was to compare trends in URiM representation from 2005 to 2020 among med-peds residents in overlapping residencies including FM, IM, and pediatrics. The secondary aim was to compare the URiM representation in these specialties to the US population.

This article was previously presented as a meeting abstract at the 2024 Academic Internal Medicine Week on April 15, 2024.

## Materials and methods

A cross-sectional analysis of race and ethnicity among med-peds, IM, FM, and pediatric residents was performed using publicly available annual reports from the National Graduate Medical Education (GME) census from 2005 to 2020 [22-37]. This data is published annually in the *Journal of the American Medical Association* and confirms self-reported race and ethnicity data jointly collected by the AAMC and American Medical Association via electronic surveys sent to program directors of accredited US residency programs. The average survey response rate was 93.4% during the study period. For this study, GME census reports were referred to by the year the residency term began (i.e., 2020-2021 is 2020). US population data was obtained through publicly available US census data. The yearly population distribution of race and ethnicity estimates was obtained from the American Community Survey, with one-year estimates from 2005 to 2020 [38].

We utilized the AAMC definition for URiM: self-identification as AA, AI/AN, NH/PI, or Hispanic [39]. Throughout all data sources, a person of Hispanic ethnicity can be of any race. Of note, the US census created a “multiracial” category in 2000 for those selecting >1 race; the GME census introduced this category in 2014. The “multiracial” and “unknown” categories were excluded from our study due to a lack of racial subcategorization within these groups.

Proportions for each group were calculated, and the Clopper-Pearson exact binomial method was used to calculate 95% confidence intervals (CIs). The Cochran-Armitage test was utilized to evaluate trends in proportions of URiM representation among residents in each specialty and the US population over the 16-year study period. Trend analysis was not included for AI/AN and NH/PI med-peds residents or NH/PI pediatric residents due to insufficient sample size for statistical analysis. However, these populations were included in the trend analysis of total URiM residents. Descriptive statistics were used to further characterize the results.

A two-tailed p-value <0.05 was considered statistically significant. A negative Z-score indicated a negative trend. SAS (version 9.4; SAS Institute, Inc, Cary, NC, US) was used for all analyses. This study was considered exempt from human subject research by the University of Texas Southwestern Institutional Review Board.

## Results

Trends in total URiM residents in med-peds and related residencies

The total number of URiM residents in all included specialties increased from 5,581 in 2005 to 8,916 in 2020 (Table 1). Overall, there was a significant positive trend in the proportion of total URiM residents from 2005 to 2020 in med-peds (p = 0.01), FM (p = 0.04), IM (p < 0.001), and pediatrics (p < 0.001). The positive trend in Hispanic residents was significant for all included specialties (p < 0.001). The AA trend was positive for FM (p < 0.001) and IM (p < 0.001). However, a negative AA trend was noted for both pediatrics (p = 0.49) and med-peds (p = 0.01), though only significant for med-peds. For NH/PI, the trend was negative and significant for both FM and IM (p < 0.001). The trend in the proportion of AI/AN residents was negative and significant for FM and IM (p < 0.001), whereas the trend was negative but not significant for pediatrics (p = 0.12).

**Table 1:** Trends in URiM Residents and the US Population From 2005 to 2020 URiM: underrepresented minorities in medicine; US: United States; CI: confidence interval; med-peds: internal medicine-pediatrics; AA: African American/Black; AI/AN: American Indian/Alaskan Native; NH/PI: Native Hawaiian/Pacific Islander *p-values reflect the results of the Cochran-Armitage test examining trends in proportions for each group from 2005 to 2020 ^†^Significant p-values are in bold ^‡^Negative Z-scores indicate a negative trend in proportions over time ^--^No p-value was calculated due to insufficient sample size for statistical analysis

	2005		2020			
	Number of residents	Percentage of population (95% CI)	Number of residents	Percentage of population (95% CI)	Two-tailed p-value for trend*	Z-score
Med-peds						
Total	1,425		1,501			
URiM	156	11.0 (9.4-12.7)	226	15.1 (13.3-17.0)	0.01^†^	2.6
AA	98	6.9 (5.6-8.3)	107	7.1 (5.9-8.5)	0.01	-2.7^‡^
AI/AN	4	0.3 (0.1-0.7)	2	0.1 (0.0-0.5)	--	--
NH/PI	7	0.5 (0.2-1.0)	1	0.1 (0.0-0.4)	--	--
Hispanic	47	3.3 (2.4-4.4)	116	7.7 (6.4-9.2)	<0.001	7.9
Internal medicine						
Total	21,885		28,677			
URiM	2,688	12.3 (11.9-12.7)	4,455	15.5 (15.1-16.0)	<0.001	6.6
AA	1,055	4.8 (4.5-5.1)	1,819	6.3 (6.1-6.6)	<0.001	7.5
AI/AN	43	0.2 (0.2-0.3)	52	0.2 (0.1-0.2)	<0.001	-4.9
NH/PI	182	0.8 (0.7-1.0)	15	0.1 (0.0-0.1)	<0.001	-17.6
Hispanic	1,408	6.4 (6.1-6.8)	2,569	9.0 (8.6-9.3)	<0.001	5.8
Family medicine						
Total	9,394		13,745			
URiM	1,550	16.5 (15.8-17.3)	2,565	18.7 (18.0-19.3)	0.04	2.1
AA	646	6.9 (6.4-7.4)	1,116	8.1 (7.7-8.6)	<0.001	4.3
AI/AN	42	0.5 (0.3-0.6)	47	0.3 (0.3-0.5)	<0.001	-5.2
NH/PI	150	1.6 (1.4-1.9)	14	0.1 (0.1-0.2)	<0.001	-19.3
Hispanic	712	7.6 (7.1-8.1)	1,388	10.1 (9.6-10.6)	<0.001	4.7
Pediatrics						
Total	7,965		9,098			
URiM	1,187	14.9 (14.1-15.7)	1,670	18.4 (17.6-19.2)	<0.001	5.2
AA	472	5.9 (5.4-6.5)	629	6.9 (6.4-7.5)	0.49	-0.7
AI/AN	21	0.3 (0.2-0.4)	21	0.2 (0.1-0.4)	0.12	-1.6
NH/PI	66	0.8 (0.6-1.1)	6	0.1 (0.0-0.1)	--	--
Hispanic	628	7.9 (7.3-8.5)	1,014	11.1 (10.5-11.8)	<0.001	9.7
US population						
Total	2.96 x 10^8^		3.31 x 10^8^			
URiM	8.38 x 10^7^	28.4	1.08 x 10^8^	32.4	<0.001	1,872.2
AA	3.78 x 10^7^	12.8	4.11 x 10^7^	12.4	<0.001	-143.5
AI/AN	2.92 x 10^6^	1.0	3.73 x 10^6^	1.1	<0.001	-209.0
NH/PI	5.27 x 10^5^	0.2	6.90 x 10^5^	0.2	<0.001	86.3
Hispanic	4.26 x 10^7^	14.4	6.21 x 10^7^	18.70	<0.001	2,476.9

Trends in race and ethnicity of med-peds residents 2005-2020

**Figure 1:**
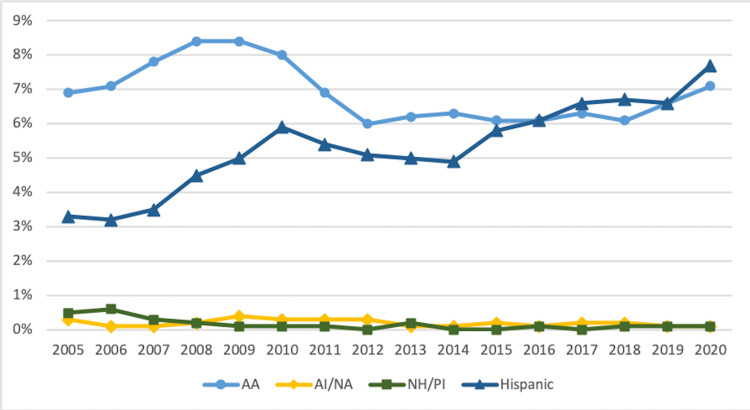
Trends in URiM Med-Peds Residents by Race and Ethnicity 2005-2020 URiM: underrepresented minorities in medicine; med-peds: internal medicine-pediatrics; AA: African American/Black; AI/AN: American Indian/Alaska Native; NH/PI: Native Hawaiian/Pacific Islander

Figure 1 displays trends in percentages of URiM med-peds residents from 2005 to 2020. There was a steady upward trend in the percentage of Hispanic med-peds residents, increasing from 3.3% (n = 47) in 2005 to 7.7% (n = 116) in 2020. The percentage of AA med-peds residents peaked at 8.4% (n = 119) between 2008 and 2009 before stabilizing at 6.0%-6.3% (n = 85-93) from 2012 to 2018 and trending up to 7.1% (n = 107) in 2020. The number of AI/AN med-peds residents remained between 0.1% and 0.4% (n = 1-5) throughout the study period, peaking in 2009. The percentage of NH/PI med-peds residents remained 0.0%-0.6% (n = 0-8), peaking in 2006.

Trends in URiM representation among med-peds and related residencies vs. US population

**Figure 2:**
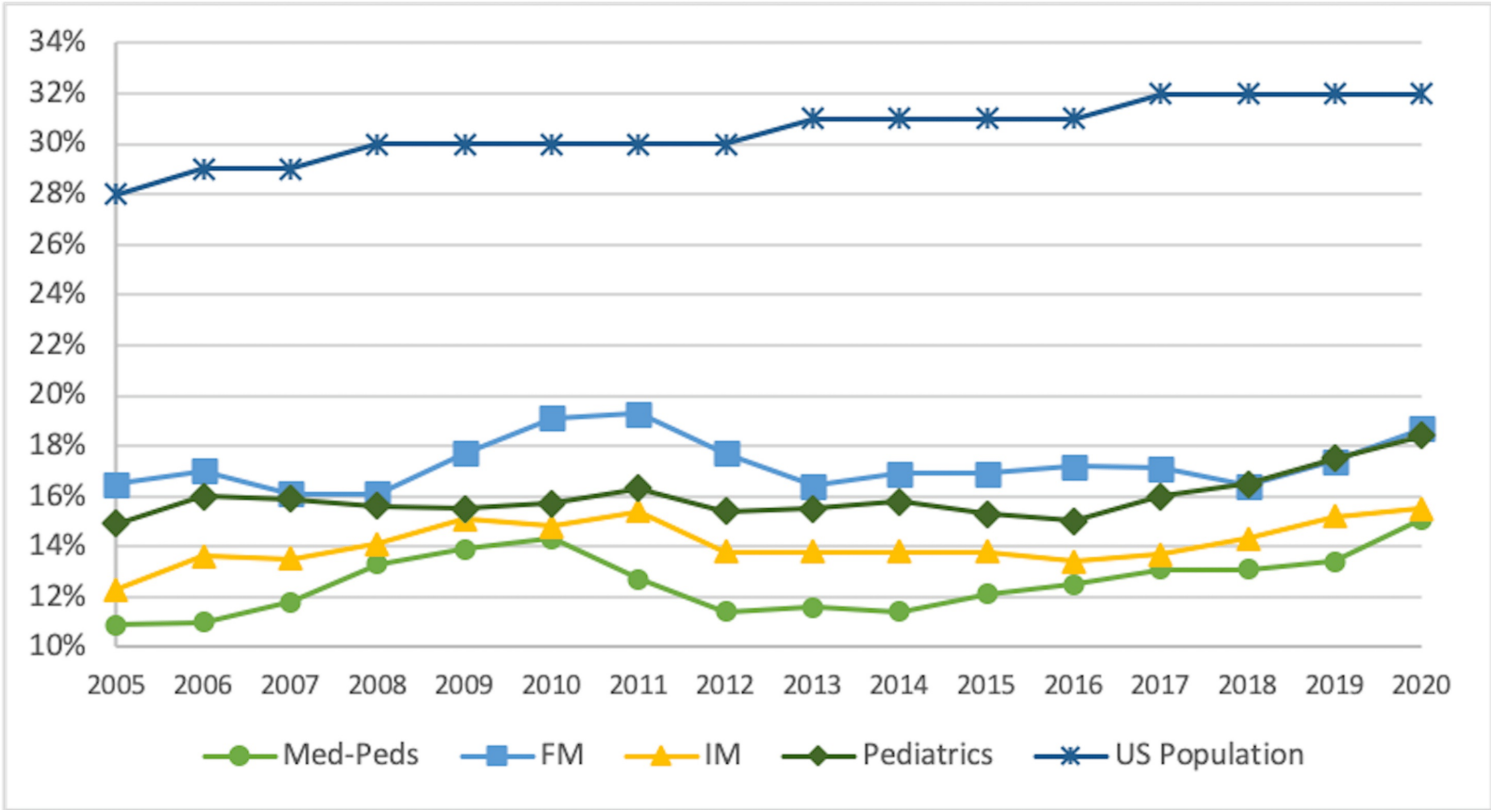
Trends in Total URiM Residents by Residency Program and Total URiM US Population 2005-2020 URiM: underrepresented minorities in medicine; med-peds: internal medicine-pediatrics; FM: family medicine; IM: internal medicine; US: United States

From 2005 to 2020, there was a significant positive trend in the proportion of the total US population identifying as a URiM race or ethnicity (p < 0.001) (Table 1, Figure 2). The trend was positive and significant for Hispanic and NH/PI (p < 0.001) populations but negative and significant for AA and AI/AN (p < 0.001) populations. However, throughout all years, the proportion of total URiM residents in med-peds, FM, IM, and pediatrics was lower than their proportion of the US population. The peak total URiM representation for med-peds, IM, and pediatric residents occurred in 2020, whereas FM peaked in 2011 (19.3%, n = 1,882). Total URiM med-peds representation remained less than FM, IM, and pediatrics throughout the study period.

**Figure 3:**
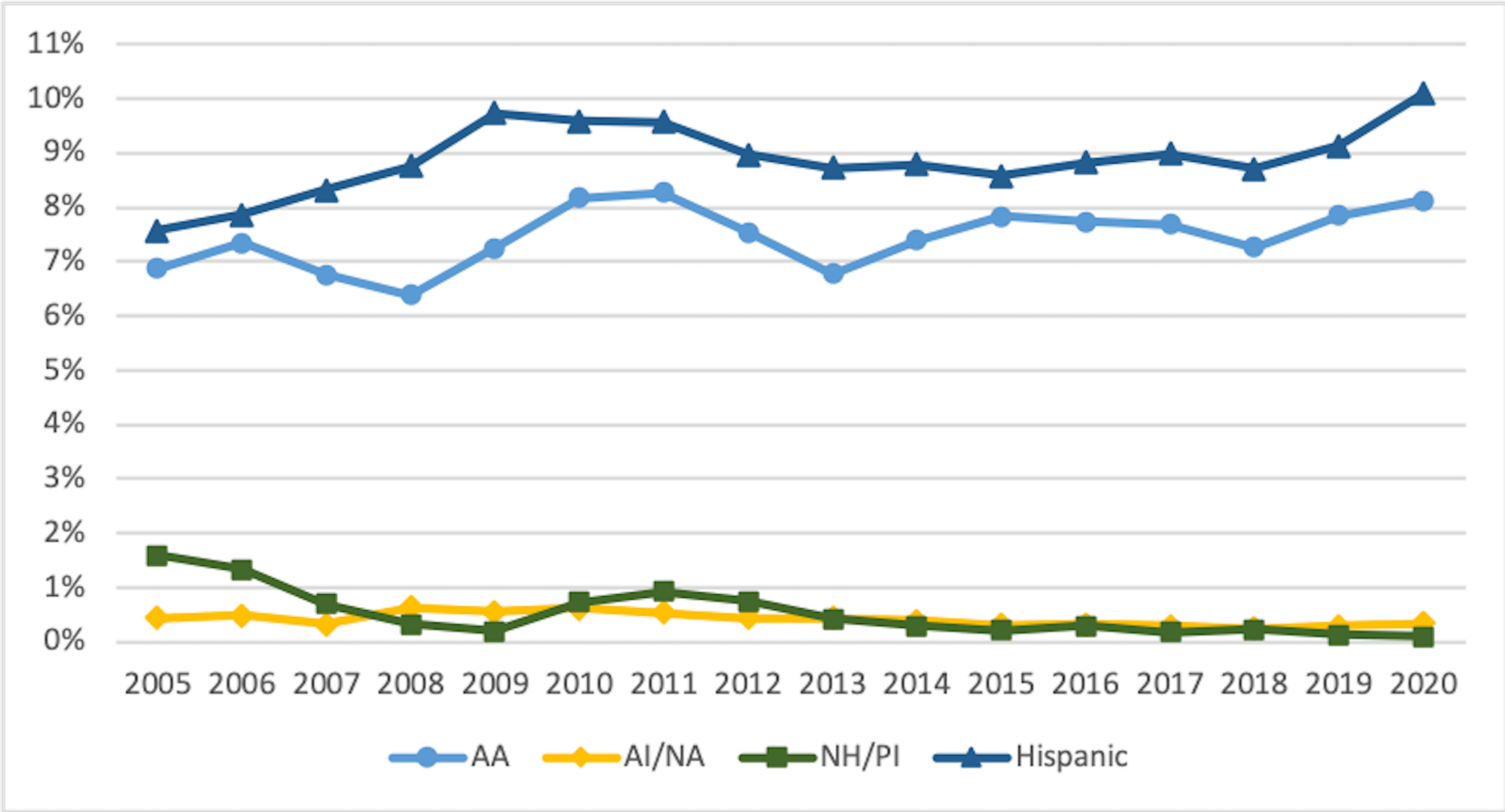
Trends in URiM Family Medicine Residents by Race and Ethnicity 2005-2020 URIM: underrepresented minorities in medicine; AA: African American/Black; AI/AN: American Indian/Alaska Native; NH/PI: Native Hawaiian/Pacific Islander

**Figure 4:**
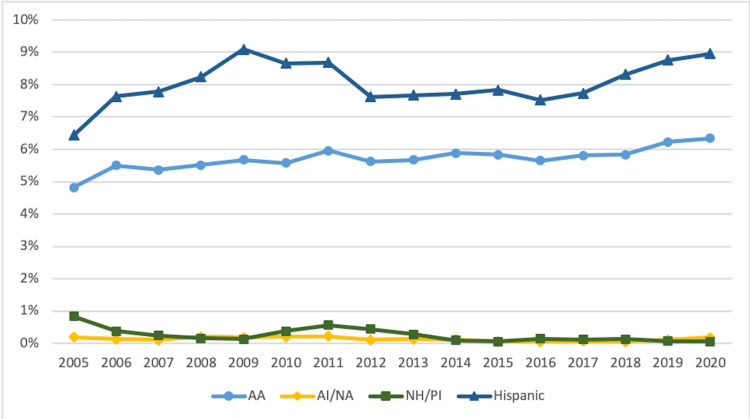
Trends in URiM Internal Medicine Residents by Race and Ethnicity 2005-2020 URiM: underrepresented minorities in medicine; AA: African American/Black; AI/AN: American Indian/Alaska Native; NH/PI: Native Hawaiian/Pacific Islander

**Figure 5:**
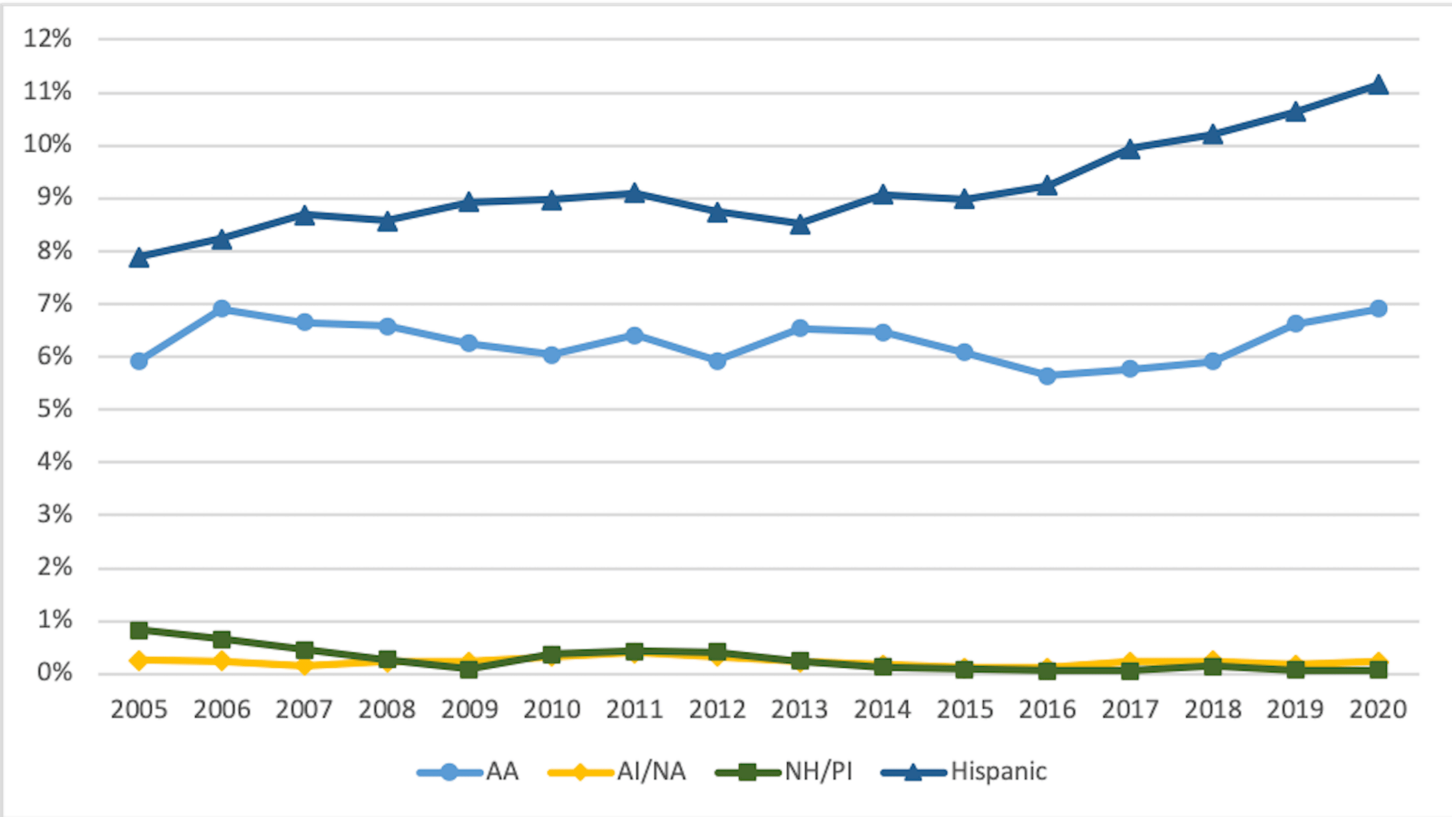
Trends in URiM Pediatric Residents by Race and Ethnicity 2005-2020 URiM: underrepresented minorities in medicine; AA: African American/Black; AI/AN: American Indian/Alaska Native; NH/PI: Native Hawaiian/Pacific Islander

Figures 3-5 display URiM trends by race and ethnicity for FM, IM, and pediatrics.

## Discussion

This cross-sectional study evaluates trends in URiM representation among med-peds and related residencies including FM, IM, and pediatrics from 2005 to 2020. In this study, we offer one of the first known comprehensive analyses of URiM representation in med-peds residencies. We found a significant positive trend in total URiM representation among med-peds residents and related specialties over the study period. However, the proportion of URiM residents in med-peds was consistently less than other analyzed specialties, which are all notably lower than the US population.

A recent study by Montez et al. examining racial and ethnic trends among pediatric residencies and fellowships utilizing GME census reports from 2005 to 2018 found no significant change in URiM proportions over time [19]. When we examined similar data with the addition of two years, there was a significant positive trend in the total proportion of URiM pediatric residents. We noted comparable positive trends in med-peds, FM, and IM. The positive trend in total URiM representation within med-peds and related residencies may reflect recent heightened efforts to increase racial and ethnic diversity in GME. In 2019, the ACGME implemented a new common program requirement to promote a diverse and inclusive workforce [12]. In addition, recent anti-racist social movements may be contributing to intensified URiM recruitment efforts across all residency training programs [40]. These initiatives may have resulted in improved residency recruitment.

Notably, these positive trends in total URiM representation did not reflect significant improvements in every racial and ethnic subgroup. The positive trend in URiM representation in med-peds was largely attributable to increased Hispanic resident representation, which was the only med-peds URiM group with a significant positive trend during the study period. Similarly, FM, IM, and pediatrics had significant positive trends in Hispanic resident representation, while trends for other URiM groups varied. Improved Hispanic med-peds resident representation was previously noted in a study by Bennett et al. examining racial and ethnic diversity within US residencies 2007-2017 [41]. There was an increase in the total US Hispanic population from 13.1% to 18.1% from 2002 to 2017 [38,42]. Though Lett et al. noted no significant change in the proportion of URiM Hispanic medical students compared to the US population over that period, the absolute number of Hispanic medical students increased in proportion to the US population, which may partially explain the positive trend in our study [42]. Unfortunately, Bennett et al. noted that at the current rate of improvement, it could take 61 years to achieve Hispanic med-peds representation comparable to the US population [41]. They also found the change in representation of AA med-peds residents from 2007 to 2017 was not significant. We found that with an additional three years of data, the significant negative trend in AA med-peds resident representation persisted. Lett et al. noted a trend toward decreased representation for AA female medical students, which may have contributed to the negative trend in AA URiM med-peds representation observed in our study [42]. However, the AA representation increased significantly in FM and IM, suggesting that a reduction in AA student enrollment does not fully explain these findings.

We noted lower total URiM representation in med-peds compared to FM, IM, and pediatrics throughout the study period. This may be multifactorial including an existing lack of diversity in med-peds residency programs. Prior research suggests that while the strongest factors influencing residency selection for all URiM residency applicants are similar to other applicants including academic reputation and resident morale, URiM applicants ranked support for minorities and diversity of the residents and faculty above other applicants [43,44]. Tiako et al. reviewed residency application data for 18 specialties for the 2019-2020 residency application cycle and found that racial and ethnic representation among practicing physicians in any specialty was positively associated with increased residency application rates among medical students [45]. Although this study excluded med-peds, these findings are likely applicable. Therefore, lower URiM representation in med-peds residencies may be exacerbated by existing lower racial and ethnic resident diversity compared to FM, IM, and pediatrics.

Exposure to med-peds and mentorship also promote consideration of a med-peds career among medical students [46]; thus, opportunities to recruit URiM medical students may be limited in geographic regions with larger URiM populations that lack med-peds programs. We noted a significant negative trend in the proportion of AA med-peds residents during our study period. While the New York City and Atlanta metro areas have the largest US AA populations, New York City lacked a consistently open med-peds program, and there were no med-peds programs in the Atlanta metro area throughout the study period [47-49]. Medical schools at Historically Black Colleges and Universities (HBCU) also lack med-peds programs. An estimated 70% of AA physicians have obtained a degree from an HBCU, and the four HBCU medical schools accounted for 14% of enrolled AA medical students in 2019 [50,51]. Additionally, there are no med-peds programs in the northwest US, Alaska, or Hawaii, which may contribute to the lack of AI/AN and NH/PI med-peds residents [47]. Establishing med-peds programs in these geographic regions and institutions may increase URiM med-peds resident representation.

URiM medical students are more likely to prioritize working with medically underserved populations and may seek residencies with an explicit focus on diversity, equity, and inclusion (DEI) [52]. Other specialties such as FM have proactively identified themselves as physicians for the underserved [53,54]. Med-peds residency programs vary in their emphasis on DEI, though there are recent initiatives emphasizing DEI in residency curricula [47,49,55]. In 2014, Johns Hopkins Medicine created a med-peds urban health residency with a curriculum focused on diversity, inclusion, and anti-racism [55]. In 2019, Icahn School of Medicine at Mount Sinai established a med-peds program with a specific aim to reduce health inequality in marginalized populations [49]. The National Med-Peds Residents’ Association recently increased its focus on DEI, including providing mentorship opportunities for URiM trainees and hosting webinars for prospective applicants to discuss DEI in med-peds [56]. Future research is needed to examine the impact of these initiatives on URiM med-peds resident representation. An evaluation of how DEI curricula vary between med-peds, FM, IM, and pediatric residencies may also be helpful.

Coordinated national efforts to improve the geographic distribution of med-peds programs and increase access to medical student mentorship are needed to increase URiM med-peds representation; national medical societies devoted to med-peds physicians can collaborate to build a multifaceted approach combining their resources. However, individual residency programs can also implement initiatives such as holistic review for all applicants, mandatory implicit bias training for faculty interviewers, and provision of financial support for early mentorship and engagement, such as attendance at national conferences hosted by URiM medical student organizations [40,57]. Unique clinical electives and second-look opportunities that increase applicant exposure have also improved URiM recruitment at some institutions and may be implemented by individual programs [58-60].

Ultimately, improved URiM representation in med-peds and all medical specialties cannot occur without increased URiM medical student enrollment. While the proportion of the US population identifying as a URiM race or ethnicity increased steadily throughout the study period, the total URiM representation in each analyzed residency remained notably lower than the US population. The persistent gap between URiM representation in these residencies and the US population may be representative of ongoing challenges in recruitment and retention at all levels of medical training. Implicit bias and racism have been shown to play a role in the admissions process throughout the entire training pathway, including undergraduate and medical school admissions [57]. Government policies including recent restrictions on affirmative action and DEI initiatives threaten current progress and create additional challenges for medical schools and residencies recruiting URiM applicants [61]. Prior statewide limitations on affirmative action were associated with reduced URiM medical school enrollment [62]. Institutions may consider adopting strategies that have successfully increased student diversity in these regions by incorporating other factors into the admissions process, such as a socioeconomic disadvantage scale [63]. Further rigorous research is needed to evaluate the broader utility of this intervention.

This study has several limitations. We were unable to evaluate trends in AI/AN and NH/PI med-peds and pediatric resident representation due to the small size of these subgroups. Future research could investigate factors influencing residency selection among these groups through targeted surveys and focus groups. In addition, we excluded unknown and multiracial residents due to a lack of available data about race and ethnicity subcategorizations within these groups. Previously published studies have cited a similar limitation [19]. The lack of the multiracial category in the GME census prior to 2014 precluded trend analysis within our study; this group was also excluded from all US census data utilized in our study. However, a portion of multiracial residents likely selected a URiM race. US census data noted that in 2020, 57.1% of the US population categorized as multiracial selected “White and some other race,” while 11.8% and 12.1% selected AI/AN or AA and another race, respectively. Thus, most of the US population categorized as multiracial may not identify as URiM [64]. The multiracial category also does not alter the number of Hispanic individuals. Despite the exclusion of the multiracial group, we noted positive trends in total URiM resident proportions in the analyzed specialties, suggesting that the inclusion of this group would likely increase the effect size and would not change this conclusion. Additionally, the finding that URiM representation is significantly below the general population in these specialties would not change. Future research detailing multiracial demographic trends would be valuable. We also did not account for trends in URiM medical student representation during this period, which could impact URiM proportions in residencies; further studies are needed to compare medical school and resident demographic trends. To avoid bias due to the unique impact of COVID-19 on medical education and widening disparity gaps, we included only data for trainees applying to residency prior to the onset of the pandemic. A specific and contextualized study is needed to understand the full impact of these events on recruitment trends in med-peds and related residencies.

Future research could also investigate the factors influencing residency and career selection among med-peds residency applicants, including a perception of increased competitiveness compared to similar programs, as well as the efficacy of current interventions to improve URiM representation. Additional research is also needed to understand trends of other underrepresented groups within med-peds and similar specialties, including lesbian, gay, bisexual, transgender, queer or questioning, and more (LGBTQ+) individuals and those experiencing disabilities.

## Conclusions

We found a significant positive trend in the total proportion of URiM residents in med-peds and related residencies from 2005 to 2020. This improved URiM med-peds resident representation was largely due to positive trends in Hispanic resident representation. Despite these improvements, URiM resident representation in med-peds and related specialties does not reflect the racial and ethnic diversity of the US population. Additionally, the proportion of total URiM med-peds residents remained below FM, IM, and pediatrics throughout the study period and should be urgently addressed.
